# Is multitasking efficient? Different metrics, different conclusions

**DOI:** 10.1007/s00426-026-02281-x

**Published:** 2026-06-01

**Authors:** Amelie C. Jung, Markus Janczyk, Dietrich Manzey, Rico Fischer

**Affiliations:** 1 Department of Psychology, University of Greifswald, Franz-Mehring-Str. 47, Greifswald, D-17489 Germany; 2 Department of Psychology, University of Bremen, Bremen, Germany; 3 Department of Psychology and Ergonomics, Technische Universitaet Berlin, Berlin, Germany

**Keywords:** multitasking efficiency, dual task, PRP, crosstalk, Time on Task

## Abstract

Multitasking—defined as the more or less overlapping execution of two or more tasks—has been shown to impair performance and induce performance costs. Nevertheless, individuals frequently engage in multitasking for private and work-related purposes. This raises the question: Do subjective impressions about multitasking efficiency diverge from objective measures, or can multitasking indeed comprise benefits in certain conditions? While previous assessments of multitasking efficiency focused mostly on central processing limitations, they largely neglected benefits of parallel peripheral task processing in overlapping task execution. To address this empirically, we conducted two multitasking studies and compared *Time on Task* (*ToT*; Reissland & Manzey, 2016), that is, the total time needed to finish both sub-tasks, in an overlapping versus a sequential dual-task condition. Experiment 1 (*N* = 18) applied a Psychological Refractory Period (PRP) paradigm with distinct task sets in Task 1 and Task 2. Experiment 2 (*N* = 32) used identical task sets to maximize between-task interference in the overlapping dual-task condition. Results of both experiments showed shorter ToT in the overlapping compared to the sequential dual-task condition, without much evidence of compensatory effects. These results suggest that the assessment of multitasking efficiency should consider the total time on task needed to perform both tasks to capture the costs of central processing limitations as well as benefits of parallel peripheral task processing. They further underscore the importance of carefully selecting and justifying the chosen evaluation metrics when assessing multitasking efficiency.

## Introduction

In our modern society, private as well as working environments are to a larger degree as ever before characterized by the need for overlapping and simultaneous engagement in more than one task at a time. There is already a good amount of evidence that humans are able to face these dynamic changes by engaging more and more in multitasking (e.g., Carrier et al., 2015; Foster, 2006; Kirchberg et al., 2015). For example, the simultaneous consumption of several media inputs has reached its peak in today’s societies (e.g., Brasel & Gips, 2011; Pilotta & Schultz, 2005). At the same time, we are confronted with warnings to refrain from multitasking by emphasizing its costs. Risking accidents by talking on the phone while driving is not only an often used example, but a real threat (Drews et al., 2008; Hancock et al., 2003; Lesch & Hancock, 2004). Some researchers attribute these burdens to an alleged inability to perform two tasks together, due to various forms of capacity limitations ranging from structural inabilities to the need to share a common capacity (Dux et al., 2006; Kahneman, 1973; Pashler, 1994; Welford, 1952). In cognitive psychological and cognitive neuroscience research, the costs of performing two tasks simultaneously have been demonstrated at the behavioral level (Pashler, 1998; see Fischer & Janczyk, 2022; Koch et al., 2018, for reviews) as well as the neural level (see Garner & Dux, 2022, for a review).

In the present study, we investigate whether increased efficiency may constitute one reason why individuals frequently engage in multitasking, despite the known costs and disadvantages. On the one hand, multitasking may create a subjective impression of increased efficiency. Engaging in multitasking can create the feeling of increased productivity, such as completing several tasks in less time compared to handling them sequentially. Such perceptions may motivate individuals to engage in multitasking, although these perceptions may not align with objective performance measures and actual costs that are observed in multitasking (Ophir et al., 2009). Thus, humans might multitask due to a cognitive fallacy overestimating their abilities and ignoring inefficiencies. On the other hand, however, multitasking may in fact *be* the more efficient mode of processing. In contrast to the entirely sequential execution of two tasks, overlapping processing can even have resource-saving benefits, for example, if some processes can run simultaneously without interfering with each other. Although initially counterintuitive, this reasoning is based on the argument that multitasking efficiency has traditionally been measured in terms of its costs without the consideration of possible benefits (Reissland & Manzey, 2016).

**Fig. 1:**
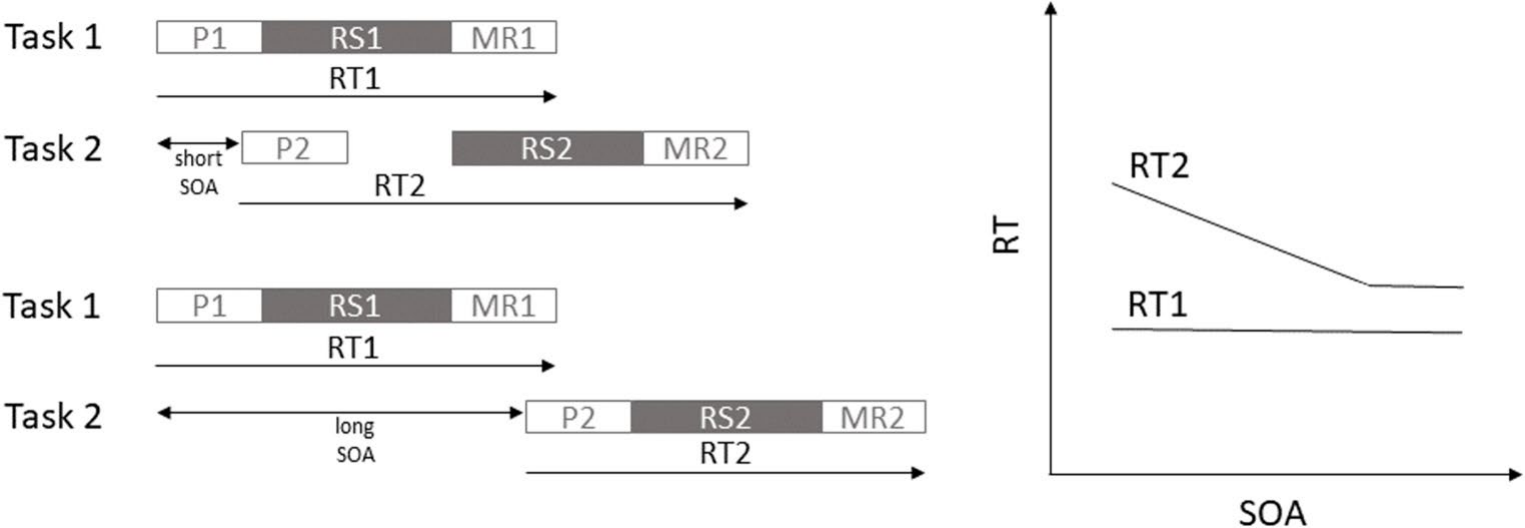
Illustration of a Response Selection Bottleneck in Dual Tasks. Task 1 and Task 2 consist of different processing stages. Peripheral stages (depicted in white) can proceed in parallel to other stages. Central response selection stages (depicted in grey) are capacity-limited by a bottleneck and, consequently, response selection in Task 2 must wait until Task 1 response selection is completed. This occurs mostly with short SOAs. At longer SOAs, response selection of Task 1 has often already finished (sometimes even before the stimulus of Task 2 is presented) and Task 2 response selection does not have to be postponed further. A typical pattern of results is shown on the right. *P* perception, *RS* response selection, *MR* motor response, *SOA* stimulus onset asynchrony, *RT* response time

In the laboratory, multitasking is often assessed in experiments where participants perform two temporally overlapping sensorimotor tasks (i.e., they perform a dual task). Dual-task costs, in terms of lengthened reaction times (RTs) and increased error rates, are either measured by comparing dual-task with single-task performance or as a result of a systematic variation of the degree of temporal overlap between both tasks (for an overview, see Koch et al., 2018). In particular, in Psychological Refractory Period (PRP) experiments, the presentation of the stimuli of two distinct tasks is separated by varying temporal intervals, typically referred to as the stimulus onset asynchrony (SOA). Whereas RTs for Task 1 (RT1) are mostly unaffected by an SOA manipulation (Pashler & Johnston, 1989; but see Strobach et al., 2015, for a discussion), Task 2 performance critically depends on the degree of temporal task overlap: RTs for Task 2 (RT2) are much longer at short compared to long SOAs and this difference is often called the PRP effect (Telford, 1931). A prominent explanation for the PRP effect is the assumption of a central capacity limitation, that is, a so-called *processing bottleneck*, that might be structural (e.g., Pashler, 1994, 1998; Pashler & Johnston, 1989; Welford, 1952) or strategic (Meyer & Kieras, 1997, 1999) in nature. For example, the *response selection bottleneck (RSB) model* (Pashler, 1994) suggests the existence of a central structural limitation, which enforces that processes of response selection can only proceed serially, that is, one at a time. In contrast, early (perceptual) and late (response execution) stages of processing may run in parallel to other processes. As a consequence, if the capacity-limited central stage of response selection is still occupied by Task 1, Task 2 response selection has to wait until its completion (Welford, 1952). Accordingly, Task 2 processing is slowed down due to the sequential nature of central response selection processing (see Fig. 1). Strategic bottleneck accounts assume that individuals have strategic control over the scheduling of task-component processing (Meyer & Kieras, 1997, 1999). Simultaneous processing, even of central stages, would be possible in principle, but participants choose to perform both tasks more sequentially. Thus, there might be a strategic benefit of processing both tasks more sequentially (see also Navon & Miller, 2002; Tombu & Jolicœur, 2002, 2003). This is not surprising, though, when the prolongation of RTs at high temporal task overlap is taken to reflect the costs of dual tasking and serves as the exclusive basis for evaluating dual-task efficiency. It has been argued that sequential task execution allows for better performance in almost every situation, as it reduces possible interference effects between tasks (Meyer & Kieras, 1997; Navon & Miller, 1987; see Fischer & Plessow, 2015, for a review), facilitates performance for difficult tasks (Fischer et al., 2007; Luria & Meiran, 2003), and comes with a reduction of the overall processing time when defined as the sum of RT1 and RT2 (Miller et al., 2009).

### Defining the efficiency of multitasking

Given that a person wants to perform two tasks, what is more efficient: Performing them in an overlapping manner, that is, processing both tasks or at least parts of it at the same time, or performing one task after the other without much overlap, that is, sequentially? When defining optimal dual-task performance in a PRP-like experiment, however, several measures of efficiency are conceivable. Miller et al. (2009) chose the sum of the RTs of both sub-tasks, the *Total Reaction Time* (*TRT*). Thus, TRT = RT1 + RT2 measures the total time it takes to complete each task and shorter TRTs indicate more efficient processing of both tasks. As shown in Fig. 1, when both tasks are performed with overlap with a short SOA, the TRT increases due to the additional time imposed by the bottleneck on Task 2 processing and hence RT2. In contrast, when the tasks are processed (more and more) sequentially with longer SOAs, the TRT becomes shorter. Accordingly, it would be more efficient to perform two tasks sequentially than with some overlap, aligning with the recommendation to avoid multitasking.

However, as Reissland and Manzey (2016) pointed out, efficiency metrics that sum RTs of the sub-tasks, such as TRT, focus exclusively on costs of capacity-limited stage processing, which may not reflect performance of the complete multitasking situation, since potential benefits of parallel processing during peripheral stages are neglected. Importantly, at a short SOA the possibility of processing peripheral stages in parallel might outweigh the costs of bottleneck processing. In addition, this potential benefit is lost when performing each task sequentially at a long SOA. To overcome this potential limitations of the TRT, Reissland and Manzey (2016) sought to define multitasking efficiency through a more holistic perspective on performance. Specifically, they proposed Time-on-Task (ToT) as an efficiency measure that was defined as the time needed for overall completion of both tasks. Hence, they suggested to take the time from the Task 1 stimulus (S1) onset until the Task 2 response (R2) is given. More precisely, for dual tasks with SOAs, ToT is calculated as SOA + RT2 (see Fig. 1). For dual tasks with a simultaneous onset of S1 and Task 2 stimulus (S2), ToT equals the longer RT, typically RT2. Finally, in a sequential task condition when the response to Task 1 (R1) triggers the onset of S2, ToT is calculated as RT1 + RT2 (and identical to TRT). Therefore, ToT considers not only the costs of overlapping central processing stages, but also potential benefits of overlapping peripheral processing stages when assessing multitasking efficiency. As can be seen in Fig. 1, ToT is shorter when SOA is short because it allows dual-task processing in an overlapping manner. In contrast, at long SOA both tasks are processed rather sequentially, resulting in a longer overall completion time.

Importantly, the alternatives in defining multitasking efficiency, that is, considering ToT versus TRT, can lead to different conclusions. To illustrate, consider an example given by Reissland and Manzey (2016): An individual works on two tasks, which both need 500 ms to process (i.e., RT1 = RT2 = 500 ms). In one condition, the tasks are presented one after the other, that is, S2 is presented only after R1. In this condition, both tasks have to be processed serially. In another condition, both stimuli are presented almost simultaneously, for example, with a short SOA of 50 ms. Further assume that the costs of the RSB amount to 200 ms. If the overall RT, as an indicator of processing efficiency, is now calculated by using TRT, the sequential processing results in TRT = RT1 + RT2 = 500 ms + 500 ms = 1000 ms, whereas overlapping processing yields TRT = RT1 + RT2 + bottleneck delay = 500 ms + 500 ms + 200 ms = 1200 ms. According to this calculation, overlapping processing is less efficient. When using ToT, in contrast, and assuming a short SOA of 50 ms, we get ToT = SOA + RT2 = 50 ms + 700 ms = 750 ms. Thus, the time needed to fully process both tasks in the overlapping condition is 250 ms shorter than in the sequential condition. In sum then, based on ToT the conclusion would be that overlapping processing is more efficient than sequential processing. Moreover, this implies that more tasks can be completed in the same amount of time during (overlapping) dual tasking compared to when performing the tasks separately and sequentially as individual tasks. Put differently, because the benefits of dual tasking outweigh its costs in an overlapping condition and since the throughput is higher then, in contrast to a sequential condition, people may engage in multitasking for this reason.

In the context of the stage logic as illustrated in Fig. 1, it appears almost inevitable that the ToT efficiency measure suggests overlapping processing as more efficient than sequential processing. Yet, at least within a PRP-like dual-task experiment, this argumentation remains theoretical, is based on illustrations with arbitrary numbers and stage durations, and disregards effects of overlapping processing other than the duration of stages. Thus, declaring overlapping processing as more efficient than sequential processing based on the theoretical logic of ToT may be premature for a number of reasons.

First, the costs resulting from bottleneck processing may be underestimated in the overlapping condition. While early central bottleneck models proposed a first-come first-serve logic that defined the bottleneck as a mere waiting period until Task 1 central processing has completed (Pashler, 1994, 1998; Pashler & Johnston, 1989), assumptions of resource sharing at a short SOA may prolong central stage processing in both sub-tasks (Lehle & Hübner, 2009; Navon & Miller, 2002; Tombu & Jolicœur, 2003), which aligns with the sometimes observed RT1 prolongation at short SOA (Strobach et al., 2015; Tombu & Jolicœur, 2002). In addition, recent research emphasized considerable executive control processes to be involved in the active scheduling of multiple task-component processing. For example, dual tasking requires the active monitoring of two distinct task streams (Damos & Wickens, 1980; Meyer & Kieras, 1997; Strobach et al., 2012) and the determination and scheduling of the task order (Darnstaedt et al., 2024; Hirsch & Koch, 2024; Kübler et al., 2018, 2019; Luria & Meiran, 2003; Strobach et al., 2021). Furthermore, executive control processes in dual tasking involve interference control to reduce between-task conflict, especially when both tasks share dimensional overlap (Fischer & Janczyk, 2022; Janczyk, 2016; Koob et al., 2023; Schuch et al., 2019) or demand the same cognitive resources (Wickens, 1984, 2002), as well as flexible processing shifts from one task set to another (Logan & Gordon, 2001; Meyer & Kieras, 1997). The suggestion that additional executive control processes are involved in overlapping compared to sequential dual-task processing corresponds well with observing neural activity in brain regions typically associated with executive functions, such as the lateral prefrontal cortex, during multitasking (Dux et al., 2006; Schubert & Szameitat, 2003; Szameitat et al., 2002). Indeed, non-invasive brain stimulation, such as transcranial direct current stimulation (tDCS) over the left dorso-lateral or posterior prefrontal cortex improved dual-task processing (Filmer et al., 2013; Mahesan et al., 2023; Manor et al., 2016). These examples illustrate that resource sharing as well as various executive control demands in the overlapping performance of two tasks may lead to an additional prolongation of central processing stages. Thus, it is conceivable that increased executive control demands that are present in the overlapping, but not in the sequential, dual-task condition render the former as less efficient, that is, producing the longer ToT.

Second, the entire argument of more efficient dual tasking in the overlapping condition than in the sequential condition, as possibly be shown with shorter ToT, is based solely on considerations of RTs. It is thus not clear whether fast overlapping processing of two tasks and the implied short ToT is accompanied by reduced accuracy. However, one can question the validity of ToT as an efficiency measure when, for example, a shorter ToT in the overlapping dual-task condition is accompanied with twice as many errors when compared with the sequential dual-task condition (for a similar critique of using only a single performance measure, see Rohrer, 1996, and Rohrer & Wixted, 1994).

Third, efficient overlapping dual-task processing as indicated by a shorter ToT might provoke further trade-offs such as higher levels of invested effort, higher perceived stress and arousal levels, or decreases in mood and subjective well-being. In fact, recent studies have shown that multitasking is associated with an increase in perceived stress that mirrors the time course of the biological stress response as assessed by increased sympathetic nervous system (SNS) activity in multitasking compared to single-task conditions (Becker et al., 2023b; see Becker et al., 2023a, for a meta-analysis).

### The present study

In this study, we tested the theoretical claim by Reissland and Manzey (2016) that performing two sensorimotor tasks in an overlapping manner is more efficient than performing them sequentially. To be clear, overlapping processing refers to temporal overlap in the processing of both sub-tasks as is typical in a PRP experiment, whereas sequential processing refers to a situation when S2 is presented as a result of giving R1. The dual-task efficiency measure ToT was calculated for RTs for the overlapping as well as the sequential dual-task condition. Accuracy, as defined by the percent of errors (PE), was calculated to control for potential speed-accuracy trade-offs (Liesefeld & Janczyk, 2019, 2023; Wickelgren, 1977).

Because it has been postulated that the efficiency of overlapping and sequential processing depends on whether the two tasks ask for the same or different resources (Wickens, 1984, 2002; see also Reissland & Manzey, 2016), we aimed to test dual-task efficiency for dual tasks with two distinct and two identical tasks in two separate experiments: Experiment 1 was a typical PRP experiment with an auditory pitch-categorization task as Task 1 and a visual number-categorization task as Task 2. Both tasks were temporally separated by two different SOAs in the overlapping dual-task condition and were performed one after the other in the sequential dual-task condition. Experiment 2 was a dual-task experiment in which both tasks consisted of identical task sets, that is, number-categorization in Task 1 and Task 2 (e.g., Fischer et al., 2007; Logan & Schulkind, 2000). This design maximized dimensional overlap between tasks. Especially in conditions of overlapping dual-task processing, increased control demands are required in order to reduce resource conflicts and/or crosstalk between tasks. Again, ToT and PE were compared between an overlapping and sequential dual-task processing condition. To assess further potential trade-offs as a consequence of reduced ToT in overlapping dual-task processing, additional measures, such as physiological and subjective arousal, subjective difficulty and effort, as well as mood and memory performance were evaluated in Experiment 2.

## Experiment 1

In Experiment 1, ToT was compared between an overlapping and a sequential dual-task condition. In the overlapping dual-task condition, a PRP procedure with an SOA of either 50 or 500 ms was applied ensuring temporally overlapping processing of components of the sub-tasks. This manipulation allowed peripheral processing stages to overlap to two different extents. Importantly, even the standard PRP instruction to respond to Task 1 before Task 2 does not prevent overlapping dual-task processing. In the sequential dual-task condition, both tasks were performed one after the other. The two tasks were distinct from each other to the extent that they employed different input modalities (i.e., auditory and visual) and different stimulus-response categorization rules (pitch categorization and odd-even number categorization). In line with the argumentation of Reissland and Manzey (2016), a shorter ToT in the overlapping dual-task condition with an SOA of 50 ms compared to the ToT of the sequential task condition would indicate multitasking benefits.

### Methods

#### Participants

We decided to test 18 participants (13 female and 5 males; 18–35 years, *M* = 22.4 years, *SD* = 4.4 years) at the University of Bremen. Thus, this sample size was similar or larger as in previous experiments with this experimental approach (e.g., Göthe et al., 2016; Johnston & McCann, 2006; Ruthruff et al., 2001; Schumacher et al., 2001). No formal a priori power analysis was conducted, but a sensitivity analysis showed that a paired *t* test (two-sided) with this sample size could detect effects of *d*_*z*_ ≥ 0.81 with a power of 1-β = 0.9 and α = 0.05. Even if applying a conservative Bonferroni correction and using α’ = 0.05/3 = 0.0167, revealed the minimum-detectable effect size as *d*_*z*_ ≥ 0.95. As will be seen, the effects of interest were larger in our experiment. All participants considered themselves right-handed. All participants gave informed consent beforehand and were rewarded for participation with course credits. According to the guidelines of the German Research Foundation (DFG), no ethics committee review was required, as the study involved only simple, non-invasive behavioral tasks with healthy participants, and did not involve any physical or psychological risks. No emotional or deceptive procedures were applied. All procedures were conducted in accordance with the Declaration of Helsinki.

#### Stimuli and apparatus

A standard PC connected to a 17-inch CRT monitor was used for stimulus presentation and response collection. The experiment was programmed in C++. Data collection took place in a dimly-lit and sound-attenuated experimental cabin at the University of Bremen. A low- (300 Hz) and a high-pitch tone (900 Hz) presented via headphones served as stimuli in the auditory-manual pitch categorization task and the digits 1, 2, 3, 4, 6, 7, 8, 9 were used as stimuli in the visual-manual number categorization task and were presented at the screen center. Two dashes, one to the left and one to the right of the screen center, served as fixation and to indicate the start of a trial. All visual stimuli were presented in white color against a black background and in font size 30. Stimuli subtended a visual angle of approximately 0.92° (height: 0.8 cm at ~ 50 cm viewing distance). Responses in the pitch discrimination task were given with the a- and s-key and those in the number categorization task with the k- and the l-key of a standard QWERTZ keyboard with the index- and middle-finger of the left and right hand. The exact stimulus-response assignment was counterbalanced across participants.

#### Procedure

The experiment comprised two tasks, the pitch and the number categorization task. Participants were required to categorize the tone stimulus as either high- or low-pitch and the number stimulus as either odd or even. Instructions emphasized fast and accurate responses in both tasks and to always categorize first the auditory stimulus (Task 1) and subsequently the number stimulus (Task 2).

Two different types of blocks were applied. In the *sequential dual-task condition*, participants were instructed to perform the auditory task and the visual task one after the other. More precisely, after the fixation signs (1000 ms), the auditory S1 was played for 50 ms. Giving the respective R1 triggered the onset of the visual S2 which remained on the screen until R2 was given or for 3000 ms. In case, the first response was given with the wrong hand or no response was registered within 3000 ms, the trial was cancelled (i.e., omitted). If applying, the corresponding error feedback was presented for 500 ms and the next trial began after an inter-trial interval (ITI) of 500 ms. Trials in the *overlapping dual-task condition* began as in the sequential dual-task condition. However, the number stimulus appeared irrespective of R1 after an SOA of 50 ms or 500 ms following the auditory S1. A trial was cancelled if not both responses were given within 3000 ms, if R2 was given before R1, or if R2 was given before S2 onset. Error feedback and ITI were as described before. Please note, that for both block types one “trial” included a pair of a tone and a visual stimulus.

The experiment started with two short practice blocks of 16 trials each, one for each condition. The experimental blocks consisted of 192 trials each. All combinations of S1, S2, and—if applicable—SOA appeared equally often in random order.

#### Data analyses

*Time on Task.* A repeated measures ANOVA with the three-level factor Condition (sequential vs. SOA 50 ms vs. SOA 500 ms) was run on ToT to assess potential benefits of overlapping over sequential task processing. As ToT reflects the total time spent on both tasks, ToT for the sequential dual-task condition is RT1 + RT2. For the overlapping dual-task condition, it is calculated as SOA + RT2 (Reissland & Manzey, 2016).

*PRP.* Paired *t* tests on RT1 and RT2 of the overlapping dual-task condition compared the two SOAs (50 ms vs. 500 ms). SOA should reveal a strong impact on Task 2 performance, with longer RT2 for short than for long SOAs (PRP effect), but only little influence on Task 1 performance.

Analyses on PE were run for completeness and in order to assess potential speed-accuracy trade-offs. To do so, wrong responses, but not misses, were counted as errors. In order to compare accuracy between all conditions (sequential vs. SOA 50 ms vs. SOA 500 ms), we analyzed PE total as the sum of PE of Task 1 and Task 2. Additionally, we investigated differences in PE between tasks separately depending on SOA (50 ms vs. 500 ms) to assess the PRP effect.

### Results

Incorrect trials and misses (7.7% of all experimental trials) were excluded before analyzing RTs. Further, trials with RTs ≤ 200 ms or RTs ≥ 2000 ms were eliminated from RT analyses (3.4% of all experimental trials). The Greenhouse-Geisser correction was applied when needed, and in these cases, the corresponding ε and uncorrected degrees of freedom are reported. Pairwise comparisons using Tukey’s correction followed-up significant ANOVA results in order to delineate the differences more precisely.

#### Time on Task

**Fig. 2:**
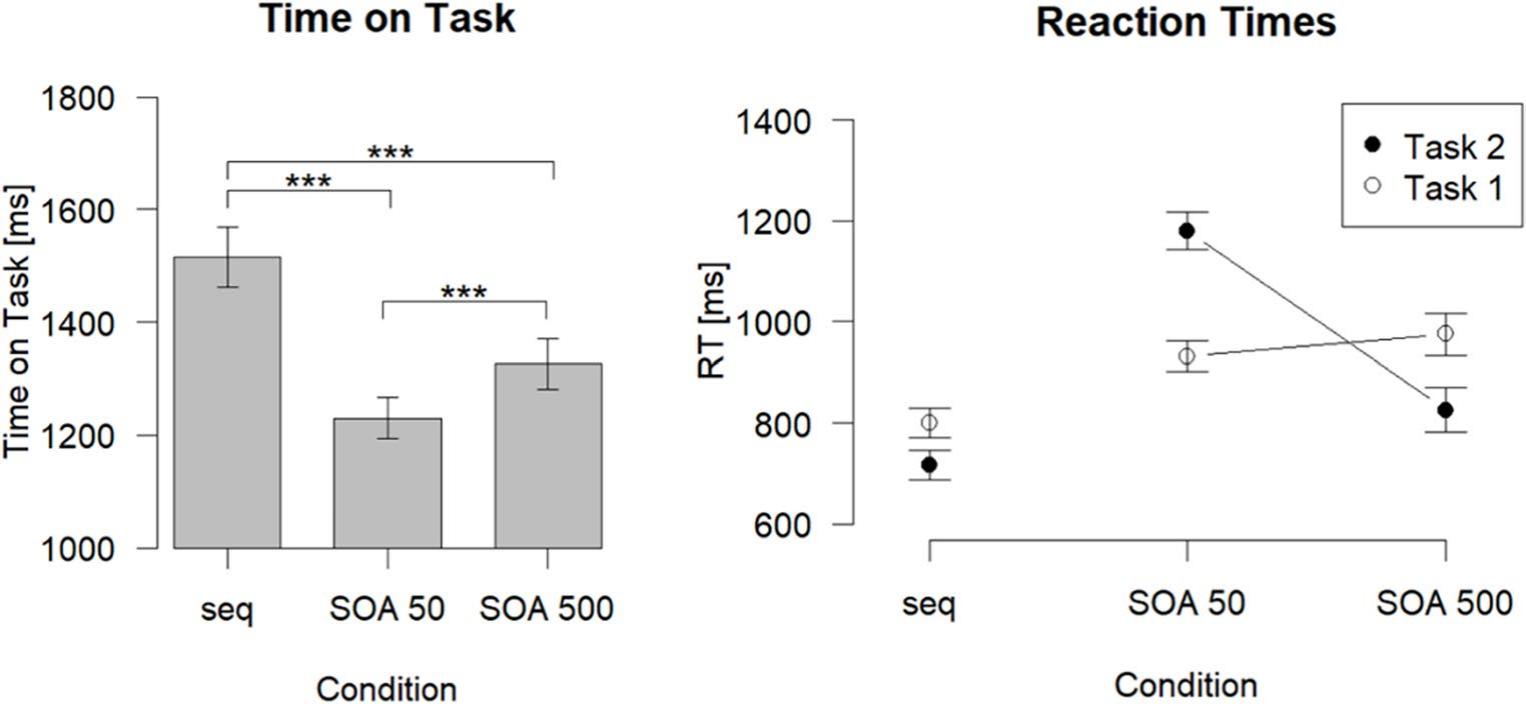
Time on Task in milliseconds (ms) as a function of Condition (left panel) and Reaction times in ms as a function of Task and Condition (right panel). Error bars represent standard errors. ***p ≤ .001, **p ≤ .01, *p ≤ .05. *seq* sequential, *SOA* stimulus onset asynchrony, *RT* reaction time

Mean ToTs are visualized in the left panel of Fig. 2. The ANOVA with the within-subject factor Condition on ToT revealed a highly significant main effect, *F*(2, 34) = 72.42, *p* < .001, η_p_^2^ = 0.81, ε = 0.74. Pairwise comparisons showed a significantly longer ToT for the sequential condition (1516 ms) in comparison to the (overlapping) condition with a 50 ms SOA (1230 ms), *t*(17) = 10.49, *p* < .001, *d*_*z*_ = 2.47, as well as in comparison to the (overlapping) condition with a 500 ms SOA (1326 ms), *t*(17) = 6.81, *p* < .001, *d*_*z*_ = 1.61. For the overlapping conditions, ToT was significantly longer for the 500 ms SOA in comparison to the 50 ms SOA, *t*(17) = -6.28, *p* < .001, *d*_*z*_ = -1.48.

PE total, reflecting the combined PE of Task 1 and Task 2, differed significantly between conditions, *F*(2, 34) = 4.97, *p* = .013, η_p_^2^ = 0.23. Pairwise comparisons revealed that PE total was significantly higher for the 50 ms SOA condition (4.9%) than for the 500 ms SOA condition (3.3%), *t*(17) = 2.62, *p* = .045, *d*_*z*_ = 0.62. The sequential condition (3.1%) did not differ significantly from the 50 ms SOA condition, *t*(17) = -2.41, *p* = .068, *d*_*z*_ = -0.57, nor from the 500 ms SOA condition, *t*(17) = -0.48, *p* = .884, *d*_*z*_ = -0.11 (see Table 1).

**Table 1:** Total (Sum of Percent Error of Task 1 and Task 2) and Task-Specific Percent Error by Condition

Condition	PE total	PE	
		Task 1	Task 2
50 ms	4.9%	4.0%	5.8%
500 ms	3.3%	0.9%	5.7%
sequential	3.1%	1.3%	4.9%

*PE* percent error

#### PRP effect

The typical PRP effect (see Fig. 2, right panel) was found as RT2 was longer at the short SOA of 50 ms (1180 ms) compared with the long SOA of 500 ms (826 ms), *t*(17) = 23.05, *p* < .001, *d*_*z*_ = 5.43. For RT1, there was also a significant difference between the SOA conditions, *t*(17) = -2.36, *p* = .030, *d*_*z*_ = -0.56, with slightly longer RTs at the long SOA (976 ms) than at the short SOA (932 ms).

PE for Task 2 did not vary significantly between the SOA conditions, *t*(17) = 0.07, *p* = .948, *d*_*z*_ = 0.02, but participants made more errors in Task 1 when the SOA was 50 ms (4.0%) compared to when it was 500 ms (0.9%), *t*(17) = 3.92, *p* = .001, *d*_*z*_ = 0.92.

### Discussion

The purpose of Experiment 1 was to investigate whether overlapping processing in comparison to sequential processing of a dual task is beneficial with regard to the overall RT when measured as ToT (Reissland & Manzey, 2016). Indeed, ToT was shorter for the two overlapping dual-task processing conditions (i.e., SOA 50 ms and 500 ms) compared to the sequential condition. This difference in ToT reflects a benefit of parallel processing of overlapping peripheral stages in the overlapping processing condition that outweighs the costs of capacity-limited stage (bottleneck) processing. In the sequential condition, by contrast, the onset of S2 was contingent upon the execution of R1, thereby precluding any possibility of parallel peripheral processing. In this condition, when the tasks were processed entirely sequential, that is, one after the other, participants took longer to respond to the whole dual task (Task 1 *and* Task 2), compared to when dual-task processing could overlap as in both SOA conditions.

Our data therefore suggest that parallel peripheral processing, due to overlapping processing stages, is beneficial and is reflected in shorter ToT. The absolute temporal benefit was substantial and amounted to 286 ms for the 50 ms SOA and to 190 ms for the 500 ms SOA condition in comparison to the sequential condition, which by adding up makes a remarkable difference in the possible task throughput within a given time. At the same time, this time-saving advantage came with no reduced accuracy for overlapping task processing.

Together, our findings show an overall processing benefit for conditions of overlapping as compared to sequential dual-task processing.

## Experiment 2

Experiment 2 served to replicate and extend the findings of Experiment 1 by including tasks with increased dimensional overlap and by assessing participants’ effort to improve the efficiency evaluation of overlapping versus sequential task processing.

First, increased dimensional overlap between tasks was realized by requiring participants to perform the same odd-versus-even number categorization on two simultaneously presented digits serving as S1 and S2 (e.g., Fischer et al., 2007; Logan & Schulkind, 2000). The same stimulus categorizations in both tasks maximize resource competition (Wickens, 2002), as well as processing interactions between tasks, so-called crosstalk. Backward crosstalk, in particular, denotes that responding to S1 is influenced by the simultaneous categorization of S2 (Bratzke & Janczyk, 2021; Hommel, 1998; Schonard et al., 2020). This can be beneficial, when both digits require the same response category (e.g., both odd) or can lead to interference, when the both digits require opposite response categories (e.g., one odd and one even). Especially in the latter case of conflict, cognitive control processes are required to shield response selection processes in Task 1 from interference of simultaneous stimulus processing. Because this crosstalk is largest with high temporal overlap of both tasks (Bratzke & Janczyk, 2021; Fischer et al., 2007; Hommel, 1998; Janczyk, 2016; Schonard et al., 2020; Schubert et al., 2008), Experiment 2 did not include an SOA manipulation, but the stimuli of both tasks appeared simultaneously.

Second, since efficiency encompasses not only the quality of task performance with regard to response speed, but also the effort required to achieve this performance level (Eason, 1963; Kahneman, 1973), we additionally assessed participants’ self-reported cognitive effort in Experiment 2. It has been argued, for example, that overlapping processing of two tasks imposes greater cognitive demands and requires increased mental effort than sequential task processing (Kahneman, 1973; Tombu & Jolicœur, 2003). Therefore, we included subjective ratings as well as physiological measures related to effortful task processing to test whether the temporal benefit of overlapping dual-task processing comes with increased effort investment. To this end, we asked participants for subjective ratings of effort and task difficulty and included ratings of arousal and mood to assess the subjective stress level in each condition block. Moreover, we examined multitasking preference and the self-rated ability to multitask, the latter one in order to explore a potential mismatch between objective performance and the subjective performance evaluation.

We further assessed effortful task processing by measuring electrocardiographic markers. On the one hand, the heart rate (HR) increases as a consequence of mental effort and investment (Kennedy & Scholey, 2000; Naccache et al., 2005; Peters et al., 1998) and reflects activity of the sympathetic nervous system and physiological stress (Becker et al., 2023a). On the other hand, mobilization of mental effort was found to be accompanied by a decrease in task-related heart rate variability (HRV), leading to a more stable heart rate (Fairclough & Mulder, 2012; Moses et al., 2007; Mulder & Mulder, 1981; Rominger et al., 2019; Wood et al., 2002). We examined HR and the root mean square of successive differences (RMSSD) serving as a measure for HRV in the time-domain.

Finally, on an exploratory level, it was tested whether retention performance in an unrelated and surprising memory task is stronger affected after the demanding overlapping dual-task condition compared to after the sequential dual-task condition. The memory task required the recognition of face stimuli after each condition. We assumed worse retention performance for the overlapping dual-task condition compared to the sequential dual-task condition, since we expected the amount of effort elicited by the former condition to have larger long-term influences.

### Methods

#### Participants

We decided to test 32 students (25 female and 7 males; 19–34 years, *M* = 24.7 years, *SD* = 3.7 years) of University of Greifswald corresponding to sample sizes of studies with a similar experimental approach (e.g., Fischer et al., 2014; Janczyk, 2016; Lehle & Hübner, 2009; Thomson et al., 2015). No formal a priori power analysis was conducted, but a sensitivity analysis showed that a paired *t* test (two-sided) with this sample size could detect effects of *d*_*z*_ ≥ 0.59 with a power of 1-β = 0.9 and α = 0.05. Even if applying a conservative Bonferroni correction and using α’ = 0.05/3 = 0.0167, revealed the minimum-detectable effect size as *d*_*z*_ ≥ 0.68. This sample comprised 27 right-handed participants. All participants gave informed consent beforehand and were compensated for participation with course credits or 6 Euro. According to the guidelines of the DFG, no ethics committee review was required, as the study involved only simple, non-invasive behavioral tasks with healthy participants, and did not involve any physical or psychological risk. No emotional or deceptive procedures were applied. All procedures were conducted in accordance with the Declaration of Helsinki.

#### Cognitive tasks

The computerized tasks were programmed and ran by E-Prime 2.0 (Psychology Software Tools, Pittsburgh, PA).

*Dual task*. Two colored dashes above and below the screen center served as fixation stimuli. The two dashes above the screen center were presented in cyan (RGB Decimal − 0, 255, 255. RGB Hex − 0x0, 0xFF, 0xFF) and those below in crimson red (RGB Decimal − 220, 20, 60. RGB Hex − 0xDC, 0x14, 0x3C). The position of the upper fixation dashes was X = 50%, Y = 47%, the position of the lower fixation dashes was X = 50%, Y = 52%, with coordinates given as percentage values relative to the screen. Throughout the experiment, the font Courier New was used at a size of 20. Stimuli subtended a visual angle of approximately 0.7° (height: 0.6 cm at ~ 50 cm viewing distance). In contrast to Experiment 1, both tasks asked for a number categorization in Experiment 2. For Task 1, the digits 2, 3, 7 and 8 appeared in blue ink in between the two dashes above the screen center. For Task 2, the red-colored digits 1, 4, 6 and 9 served as stimuli and were presented between the two dashes below the screen center. Key assignment was the same as for Task 2 in Experiment 1.

In both, the overlapping and the sequential dual-task condition, a trial consisted of Task 1 and Task 2 and participants were asked to respond as fast and as accurately as possible first to Task 1 and only then to Task 2. After the completion of the two tasks, participants were required to initiate the onset of the next task pair with a separate button press (space bar). In the sequential dual-task block, each trial began with the presentation of the fixation dashes (1000 ms) and was followed by S1 onset, which remained for 3000 ms or until response. S2 onset was prompted with R1 and it remained visible for 3000 ms or until R2 was given. The fixation dashes remained on the screen during stimulus presentation. In case of an error, the German word “falsch” (wrong) was presented for 500 ms as feedback. A correct response was followed by a blank screen and participants were instructed to press the space bar to initiate the subsequent trial sequence. The blank screen remained for 500 ms or until keypress. If the space bar was not pressed within this time frame, a warning message saying “Leertaste zu langsam gedrückt” (space bar pressed too slowly) was shown for 2000 ms. In the overlapping dual-task block, each trial started again with the presentation of the fixation dashes for 1000 ms, followed by S1 and S2, which appeared simultaneously. Stimuli remained for 3000 ms or until responses were given. Feedback messages and initiation of the next trial were the same as for the sequential condition. The experimental blocks consisted of 192 paired trials each, 384 in total.

*Memory task*. For the face-recognition task, 160 pictures of young/old and female/male faces taken from the Glasgow Unfamiliar Face Database (Burton et al., 2010) served as stimuli. In the encoding phase, the participants were presented with 40 consecutive pictures. Each stimulus was shown in the center of the screen for 1000 ms with a time interval of 500 ms between each stimulus. In the recognition phase, when either the overlapping or the sequential dual-task block was completed, the 40 previous and 40 new faces were presented and participants should rate each face as either familiar (q-key using the left hand) or unfamiliar (p-key using the right hand). Each stimulus was presented in the center of the screen for 3000 ms or until response. This whole procedure was repeated with 80 new face stimuli after the counterbalanced block type was also finished.

#### Subjective measurements

*Multitasking preference*. We used a translated version of the Multitasking Preference Inventory (MPI; Poposki & Oswald, 2010) to assess participants’ preference for multitasking. The questionnaire comprises 14 items describing single- and multitasking situations. Participants state how much they (dis)agree with each statement on a 1-to-5 Likert-scale (“strongly disagree” to “strongly agree”). The MPI score is the sum of all items, reaching from 14 (minimum) to 70 (maximum). The larger the score, the higher the preference for multitasking.

*Multitasking ability*. Participants reported their estimated multitasking ability by answering the question: “Wie gut, denken Sie, sind Sie im Multitasking?” (“How good, do you think, you are at multitasking?”). They were required to indicate their answer by marking a position on a 10 cm visual analog scale, ranging from 0 (not good at all) to 100 (very good).

*Affect*. The German version (Zwosta et al., 2013) of the Affect Grid (Russell et al., 1989) served as a questionnaire to let participants rate their current mood. Specifically, the single item (“Please rate how you are feeling right now.”) is answered by placing a checkmark somewhere within the grid. The grid consists of one horizontal displeasure-pleasure dimension and one vertical sleepiness-arousal dimension. Both scales range from 1 to 9. For the affective valence rating, higher scores indicate pleasant feelings. For the arousal rating, higher scores indicate higher arousal.

*Effort and Difficulty*. Participants rated how effortful and how difficult the just performed block had been using two visual analog scales reaching from “not at all effortful/difficult” to “very effortful/difficult”. They were asked to draw a checkmark somewhere on a 10 cm line to indicate their rating for perceived effort and perceived difficulty.

#### Physiological measures

ECG measurements were conducted using an eMotion Faros 180° biosignal recorder (1000 Hz sampling rate) by Bio Sign GmbH and Ambu^®^ BlueSensor M electrodes. Specifically, two electrodes were attached below the right and the left clavicle bone each. Another electrode was placed below the left rip bone.

#### Procedure

The experiment was run as a lab study and took about 1 h per participant. First, participants read and signed the informed consent. Then the ECG electrodes were attached to the participants. After being seated, participants filled out a questionnaire asking for their demographics and the MPI (Poposki & Oswald, 2010). Further, they rated their multitasking ability and stated in a short-written format their reasons for multitasking. Subsequently, participants were requested to rate their current mood using the Affect Grid (Russell et al., 1989) for the first time. This served as a baseline measurement of affective valence and arousal. After that, participants were introduced to the behavioral task by working on 16 overlapping and sequential dual-task trials respectively. This was followed by a baseline ECG measurement which lasted 5 min. During this time, participants were requested to relax and just do nothing. After the ECG baseline measurement, heart rate measurements were taken continuously. Since only cardiovascular data collected during overlapping or sequential dual tasking was used for analyses, exact time points of the task’s start/end were documented by the experimenter and also extracted from the E-Prime data file.

The first part of the experiment started with the memory task and participants were asked to view and memorize 40 different faces for a later recognition test. The face list was followed by either the overlapping or the sequential dual-task block. At the end of this block, participants again rated their current mood and evaluated how effortful and how difficult the last block had been. Then, the memory recognition test followed, in which participants had to categorize 80 faces as either familiar or unfamiliar. The second part of the experiment included a repetition of all these steps (including the memorization of 40 new faces). Thus, each participant underwent both block types (overlapping and sequential), with the order of block types being counterbalanced between participants. Afterward, participants were asked, which of the two experiment parts they found more pleasant and why.

#### Data analyses

Paired *t* tests were performed to compare the effect of Condition (sequential vs. overlapping) on RTs and PE of both tasks as well as ToT and PE total. In general, ToT was defined as RT1 + RT2 in the sequential dual-task condition and as RT2 in the overlapping dual-task condition, since there was no SOA and stimuli were thus presented simultaneously (and R2 was never given before R1). In addition, we considered Initiation Time defined as the time it took from R2 execution to initiate the next trial onset by pressing the space bar. If the space bar was not pressed within 500 ms, the trial was classified as an initiation error. The percentage of initiation errors was then compared between conditions.

Preprocessing cardiovascular data and computing HR and RMSSD was done with the MATLAB (The MathWorks Inc.) application HRVTool (Vollmer, 2019). In case an RR interval (i.e., the time between two successive R waves representing ventricular depolarization) could not be identified by the algorithm accurately, manual corrections were applied. Then, cardiovascular data were analyzed by running repeated measures ANOVAs including the three-level factor Condition (sequential vs. overlapping vs. baseline).

For the recognition task, *d’* was calculated as *z*(hit rate) – *z*(false alarm rate) and subsequently compared between conditions using a paired *t* test.

Subjective ratings of task difficulty and perceived effort were analyzed using paired *t* tests on the scores (1-100) of the visual analog scale. Arousal and valence ratings were analyzed with a repeated measures ANOVA including the factor Condition (sequential vs. overlapping vs. baseline).

### Results

#### Behavioral data

**Fig. 3:**
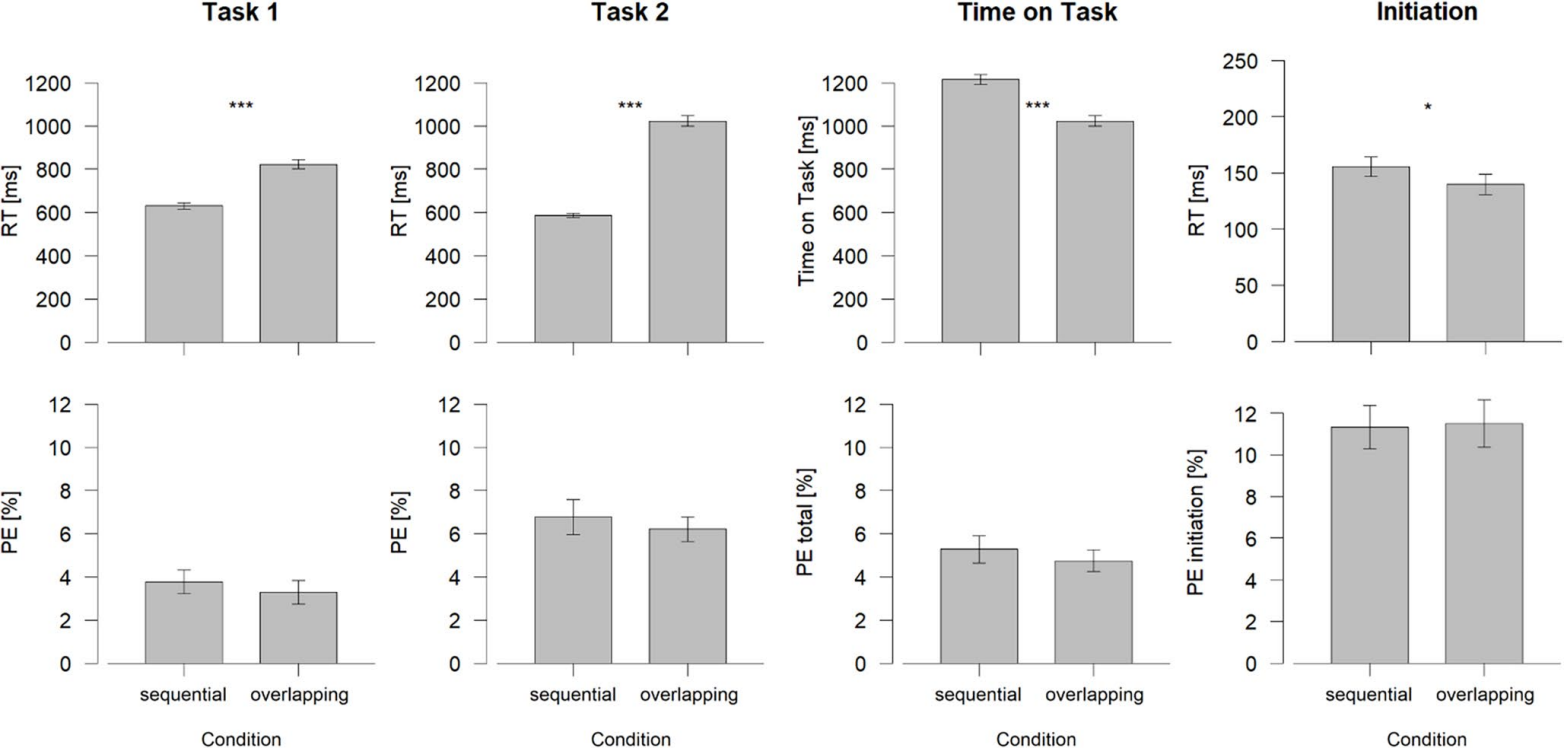
Reaction Times in milliseconds (ms) and Percent Errors for Task 1 and Task 2, Time on Task, and Initiation Time as a Function of Condition. Error bars represent standard errors. PE total equals the sum of percent error of Task 1 and Task 2. ***p ≤ .001, **p ≤ .01, *p ≤ .05. *RT* reaction time, *PE* percent error

Incorrect trials (8.5% of all experimental trials) were excluded before analyzing RTs. Further, trials with RTs ≤ 200 or RTs ≥ 2000 ms were discarded for RT analyses (1.7% of all experimental trials). Results are presented in Fig. 3. The Greenhouse-Geisser correction was applied when needed, and in these cases, the corresponding ε and uncorrected degrees of freedom are reported. Pairwise comparisons using Tukey’s correction followed-up significant ANOVA results in order to delineate the differences more precisely.

As in Experiment 1, we found a significantly longer ToT for the sequential (1215 ms) compared to the overlapping dual-task trials (1023 ms), *t*(31) = -12.77, *p* < .001, *d*_*z*_ = -2.26. The *t* test on RT1 revealed also a significant difference between conditions, *t*(31) = 13.33, *p* < .001, *d*_*z*_ = 2.36. Here, RT1 was longer in the overlapping (822 ms) than in the sequential dual-task block (629 ms). Additionally, RT2 was also longer in the overlapping (1023 ms) compared to the sequential dual-task block (586 ms), *t*(31) = 27.75, *p* < .001, *d*_*z*_ = 4.90. Lastly, initiation of the next trial differed significantly between conditions, *t*(31) = -2.52, *p* = .017, *d*_*z*_ = -0.45. The initiation time was shorter for overlapping (140 ms) than for sequential dual-task trials (156 ms).

The *t* tests on PE did not reveal significant effects, all *t*’s ≤ -0.80 and *p*’s ≥ 0.427.

#### Cardiovascular data

**Fig. 4:**
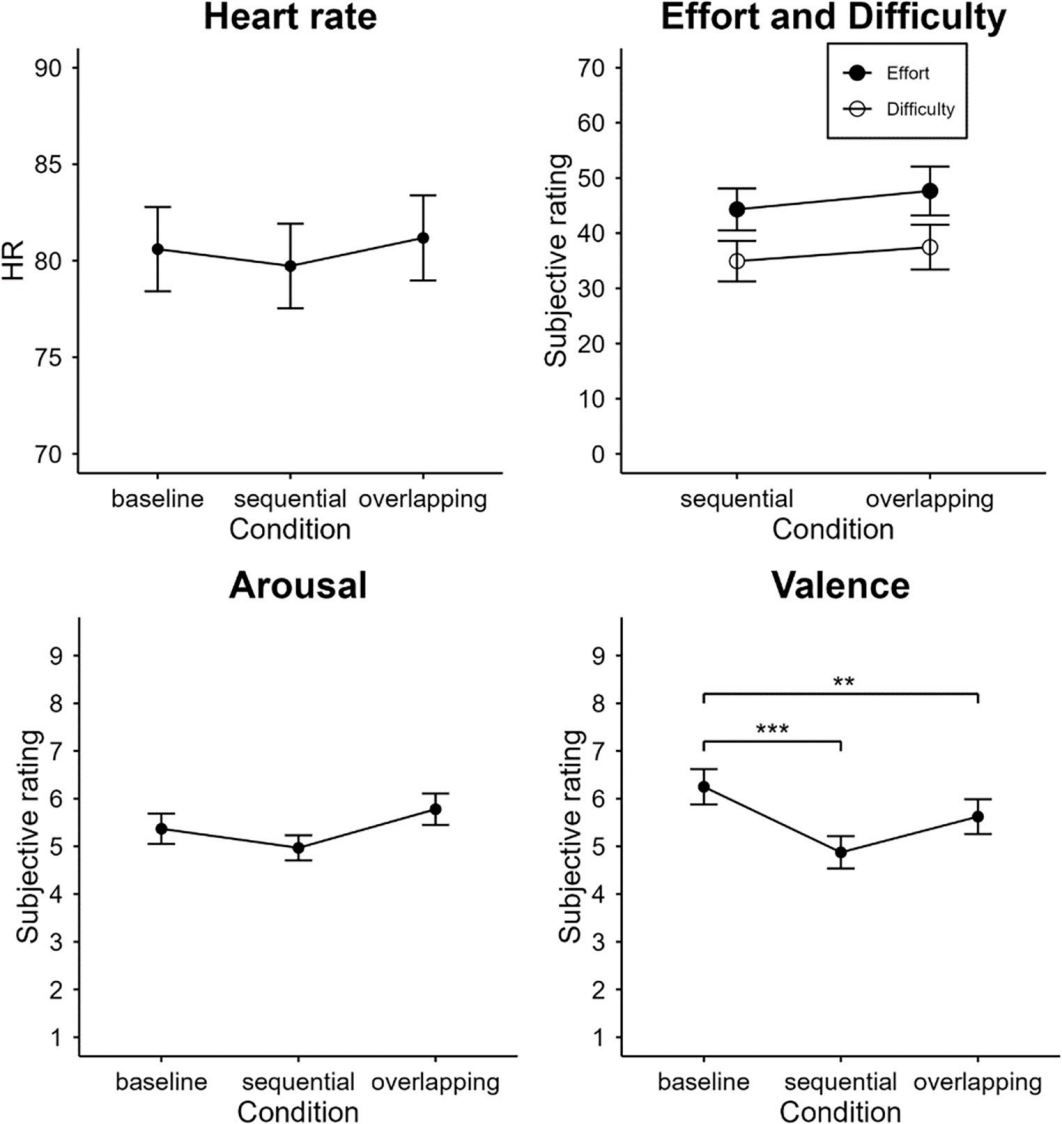
Heart Rate and Subjective Ratings as a Function of Condition. Error bars represent standard errors. ***p ≤ .001, **p ≤ .01, *p ≤ .05. *HR* heart rate

Heart rate did not differ between conditions, *F*(2, 62) = 2.34, *p* = .119, η_p_^2^ = 0.07, ε = 0.78 (see Fig. 4). RMSSD was not affected by Condition either, *F*(2, 62) = 0.95, *p* = .392, η_p_^2^ = 0.03.

#### Recognition task

Contradicting our hypotheses, recognition performance did not differ between conditions, *t*(31) = -1.58, *p* = .125, *d*_*z*_ = -0.28 (after sequential: *d’* = 0.77, after overlapping: *d’ =* 0.95).

#### Subjective ratings

Ratings of task difficulty and perceived effort did not reveal significant differences, *t*(31) = 0.98, *p* = .335, *d*_*z*_ = 0.17 and *t*(31) = 0.87, *p* = .392, *d*_*z*_ = 0.15, for difficulty and effort ratings, respectively.

There was no overall significant difference between arousal ratings, *F*(2, 62) = 2.71, *p* = .074, η_p_^2^ = 0.08. The ANOVA on valence ratings revealed a significant effect of Condition, *F*(2, 62) = 13.97, *p* < .001, η_p_^2^ = 0.31. According to the pairwise comparisons, there was no difference between the sequential (4.9) and the overlapping dual-task conditions (5.3), *t*(31) = 1.47, *p* = .319, *d*_*z*_ = 0.26. However, both of them showed lower ratings than the baseline condition (6.5); baseline versus sequential condition: *t*(31) = 4.85, *p* < .001, *d*_*z*_ = 0.86, baseline versus overlapping condition: *t*(31) = 3.54, *p* = .004, *d*_*z*_ = 0.63 (see Fig. 4).

Finally, 19 out of 32 participants rated the overlapping dual-task block as more pleasant compared to the sequential dual-task block.

#### Multitasking preference and ability

We found an average MPI score of 37.3 (*SD* = 10.0). The overall mean of subjectively rated multitasking ability was 42.3 (*SD* = 22.6). Thus, multitasking preference as well as multitasking ability were at medium level in our sample. None of the subjective ratings correlated with ToT in the overlapping dual-task condition, ability: *r*(32) = -0.25, *p* = .176, preference: *r*(32) = -0.11, *p* = .543.

### Discussion

Experiment 2 served to further investigate a potentially higher efficiency of overlapping over sequential dual tasking by implementing two additional features: (1) Crosstalk between the two tasks at conditions of high temporal overlap and (2) subjective and physiological measures of experienced effort during overlapping and sequential dual tasking.

At the behavioral level, we again found a shorter ToT for the overlapping dual-task condition. That is, although individual RTs for each task were longer in the overlapping condition than in the sequential condition, the total time to complete both tasks was significantly shorter when performed in an overlapping way. Remarkably, the higher risk for crosstalk between tasks due to dimensional overlap did not reduce the temporal benefit associated with overlapping dual-task processing. In addition, the faster initiation of the next trial-pair in the overlapping condition further supports the conclusion that overlapping task processing enabled participants to complete the whole block earlier. Despite the simultaneous stimulus presentation (SOA = 0 ms), and thus the maximization of temporal task overlap, the temporal benefit of ToT in the overlapping condition was not accompanied with an increased PE.

Participants reported no overall differences in arousal ratings following the baseline, the overlapping or the sequential dual-task block. Furthermore, we did not observe significant differences in the cardiovascular markers (HR and HRV), subjective ratings of effort and task difficulty, or the recognition performance between the dual-task conditions. Thus, in the present setting we could not find evidence that overlapping dual-task processing may cost participants considerably more effort than the sequential processing–neither in terms of mental investment nor physiological load (see General Discussion section for a more thorough discussion). Finally, roughly 60% of the participants (*n* = 19) rated the overlapping dual-task block as more pleasant than the sequential dual-task block, aligning with the descriptive results of the valence ratings.

## General discussion

In the present study we aimed to compare the efficiency when processing two tasks either in an overlapping or a sequential manner. While previous studies have shown that sequential processing of the two sub-tasks–rather than overlapping processing–represents the optimal strategy for efficient dual tasking (Miller et al., 2009), we argued that the applied measure of dual-task efficiency (i.e., TRT) focused solely on central processing limitations, but neglected potential advantages of parallel peripheral stage processing. In contrast to the TRT efficiency measure, which simply sums the RTs of both tasks, the ToT measure considers the total time from S1 to R2. This approach captures both, the costs associated with central processing limitations and the potential benefits of overlapping processing at peripheral stages.

Experiment 1 combined an auditory pitch categorization and visual number categorization in a PRP experiment. In the overlapping dual-task condition, the stimuli of both tasks were presented with varying SOAs (50 and 500 ms), whereas in the sequential condition, S2 appeared when R1 was executed. Experiment 2 incorporated a dual-task experiment with dimensional overlap and simultaneous stimulus presentation (SOA = 0 ms) to increase crosstalk between both (number categorization) tasks. The results were straightforward: The ToT in both experiments and the initiation time (Experiment 2) were shorter in the overlapping dual-task condition, indicating more efficient dual-task processing compared to the sequential condition.

In both the PRP experiment (Exp. 1) and the dual-task experiment with simultaneous stimulus onset (Exp. 2), the executive demands on coordinating central task processing did not lengthen processing to a degree that it exceeded the temporal benefits of overlapping (peripheral) processing. In addition, the benefit of the overlapping dual-task condition was not accompanied by a decrease in accuracy. Importantly, using the simultaneous stimulus presentation (SOA = 0 ms) in Experiment 2 and maximizing temporal task overlap, did not lead to increased error rates. Therefore, the present data do not suggest that the time-saving processing in the overlapping dual-task condition is the result of a mere speed-accuracy trade-off (e.g., Heitz, 2014; Liesefeld & Janczyk, 2019, 2023; Wickelgren, 1977).

Recent research suggests that multitasking can lead to larger perceived stress and an increase of the biological stress response (as measured by SNS activity) compared to single tasking (Becker et al., 2023a, 2023b). However, the results of the subjective arousal and valence ratings, subjective effort and difficulty ratings, as well as the obtained cardiovascular markers (i.e., HR, HRV) in Experiment 2 do not support this reasoning. Furthermore, also the performance of the recognition task used as a memory loading task did not differ dependent on whether participants performed the dual task in the overlapping versus sequential condition. This might be taken as an indication that overlapping processing did not put higher demands on memory than sequential processing. However, it cannot be ruled out that the lacking effects in objective stress responses and retention performance were just due to the use of a dual task consisting of two relatively simple categorization tasks which only demand few memory resources. Thus, it cannot be excluded, that performing more complex and demanding dual tasks in an overlapping compared to a sequential manner, might be associated with increased physiological costs as well as declines in memory performance (see, e.g., Kunde et al., 2012).

On the basis of ToT as the efficiency measure, we obtained better dual-task performance when both tasks were performed in an overlapping manner compared to sequentially without trade-offs in invested effort or perceived increases in difficulty. At first glance, this appears at odds with a study by Lehle et al. (2009), who found that overlapping (parallel in their terminology) dual-task processing was preferred over serial processing, as it demanded less mental effort despite resulting in poorer performance. However, Lehle et al. (2009) applied an instruction manipulation to perform two tasks either with equal emphasis (i.e., in parallel) or with priority on Task 1 over Task 2 (i.e., more serially) to manipulate the extent of crosstalk between two tasks (Lehle & Hübner, 2009; Plessow et al., 2017). Importantly, their study did not include a sequential dual-task condition, as both the parallel and the serial instruction consisted of a dual-task condition, in which stimuli from both tasks were presented simultaneously (i.e., SOA = 0 ms). Furthermore, poorer performance was seen in longer RTs and higher PE especially for Task 1 in the parallel compared to the serial instruction condition.

Again, this emphasizes that evaluating dual-task efficiency requires a careful selection and justification of the efficiency measure, as this choice can lead to substantial differences in the outcome and its interpretation. More specifically, the conceptualization of assessing dual-task performance in terms of the sum of the RTs of both tasks (as in TRT) versus considering the overall time of finishing both tasks (as in ToT), should be driven by the specific purpose of the research. The TRT measure provides specific insights in the time costs involved in conflicts between tasks on the central processing stage. Thus, this measure should be chosen if one wants to specifically assess the impact of different task or context variables on these time costs. It is this perspective which makes this “dual-task efficiency” measure particularly suited to PRP research, which often focuses on an understanding of the central bottleneck. However, this measure is less suited if one wants to assess dual-task efficiency in terms of overall task performance, that is, if one wants to investigate to what extent it might be beneficial to work on two tasks in an overlapping versus a sequential way. This latter purpose is, we would argue, better served by using the ToT measure, as it considers not only costs, but also benefits of overlapping task-component processing and, thus, reflects the net performance effects of multitasking (Reissland & Manzey, 2016). However, even the ToT measure limits the perspective on just assessing dual-task efficiency in the time domain. For a complete assessment of dual-task efficiency in terms of overall task performance, it seems important to include both, RT and accuracy measures, as well as further assessments of subjective and (possibly) objective effort investment to inform about potential performance trade-offs.

Returning to the question of why people multitask, the ToT efficiency measure suggests that individuals are indeed faster when performing two tasks overlapping rather than sequentially. Importantly, the performance benefit is not accompanied by any trade-offs with respect to accuracy or effort investment. Thus, a preference for multitasking may not be a cognitive fallacy, but may reflect an objective performance benefit when considering the total time it takes to perform two tasks, while it still needs to be investigated whether participants are actually aware of the potential time savings. Nevertheless, the present data should not be interpreted as a general recommendation in favor of overlapping multitasking, as they are based on rather simple dual tasks (Fischer & Janczyk, 2022). Certainly, it needs to be determined to which extent more complex task combinations yield the same results. For example, the extent and level of resource conflicts and possible crosstalk between tasks should be varied (e.g., task demand levels, response modalities) to examine whether the temporal benefit of overlapping processing still holds. Moreover, real-world multitasking scenarios often involve substantially higher risks and complexity than laboratory tasks. While the results of Experiment 2 showed no clear preference for multitasking in our sample, such scenarios show that people continue to engage in multitasking despite detrimental effects of driving while using a cell phone for example. In addition, it remains to be tested whether extended periods of overlapping dual tasking may eventually lead to increased effort and fatigue.

## Data Availability

Research data are available at PsychArchives, https://doi.org/10.23668/psycharchives.21785.
